# The Symptoms and Treatment of Acute Follicular Tonsillitis

**Published:** 1957-04

**Authors:** S. G. Flavell Matts

**Affiliations:** Resident Medical Officer Royal Devon and Exeter Hospital


### THE SYMPTOMS AND TREATMENT OF ACUTE FOLLICULAR TONSILLITIS

BY

S. G. FLAVELL MATTS, M.B. (B'HAM), M.R.C.S.

*Resident Medical Officer Royal Devon and Exeter Hospital*

Acute follicular tonsillitis is quite common and rarely justifies admission to hospital. Perhaps for this reason its treatment differs widely from one doctor to another. It seems still quite common practice to use sulphonamides, in spite of the work of Landsman, Grist, Black, McFarlane, Blair and Anderson[1] and Macdonald and Watson,[2] who showed that sulphonamides were not effective in the treatment of streptococcal tonsillitis. Denny, Wannamaker, Hahn[3] gave 600,000 units of procaine penicillin daily for 5 days and concluded that it relieved the sore throat and headache, but had no effect on chilliness. Penicillin is also beneficial in the prevention of recurrences in people who have already had rheumatic fever (Massell, Sturgis, Knobloch, Streeper, Hall, and Norcross).[4]

However it seems important to know if the routine use of either penicillin or sulphonamides is justified in the ordinary uncomplicated case, in the absence of a history of rheumatic fever, and it was to this end that this small but carefully controlled series was studied.

### PATIENTS

In all sixty patients were studied. They were all serving airmen who had been examined on entry into the R.A.F. and no evidence of chronic E.N.T. disease had been discovered. Their ages ranged from 18–45 years.

All patients were admitted to the station Sick Quarters ward, all were seen and followed up and assessed once or twice daily. Clinical methods were used to pronounce the patient "cured" and in all cases symptoms and signs had to have resolved before the patient was so considered. The patients were not allowed to leave before cured and no patient was informed what treatment he was receiving. There were no bacteriological investigations.

All cases were typical of the picture of acute follicular tonsillitis, the appearances being as follows:

The tonsils were enlarged, reddened, oedematous, with the tonsillar crypts filled with yellowish or greyish exudate giving the tonsils the appearance of being covered with specks; sometimes this progressed later to a continuous exudate covering most of the tonsils. The pharynx was always inflamed to a certain extent and some degree of cervical adenitis invariable.

In addition some, or all, of the following symptoms were present: general malaise, headache, sore throat, dysphagia of varying severity, aches and pains in other parts of the body, severe continuous pain in the throat, shivering, nausea, extreme weakness, foetor of breath, dizziness.

Of the 60 cases 92 per cent. presented with some fever and in 64 per cent. it was above 100° F.

About one half of the patients said they had had a previous attack of tonsillitis. Only 12 per cent. knew of any possible source of infection.

Table 1 shows the relative frequency of these presenting symptoms expressed as percentages.

TABLE I

PRESENTING SYMPTOMS IN SIXTY PATIENTS WITH TONSILLITIS

| Presenting Symptom | Percentage of all cases in which present |
|---|---|
| Sore Throat | 84 per cent. |
| Dysphagia | 66 ,, ,, |
| Headache | 56 ,, ,, |
| Extreme Weakness | 8 ,, ,, |
| General Malaise | 56 ,, ,, |
| Shivering | 24 ,, ,, |
| Nausea | 6 ,, ,, |
| Pains and Aches in back, limbs or joints. | 12 ,, ,, |
| Aching and Swelling in neck | 8 ,, ,, |
| Dizziness | 8 ,, ,, |
| Black out or Fainting | 4 ,, ,, |

The treatment was determined by selection of alternate cases.

All patients were given the following "symptomatic" treatment: Tabs. cod. co 2, six hourly. Warm gargles of pot. chlor. and phenol, q.d.s. They were given a light diet and increased fluids.

In addition there was added in those cases that happened to be selected either sulphamezathine 6–8g. daily in 4-hourly doses—penicillin distaquaine 600,000 units b.d. by I.M. injection, or a combination of the two.

One patient developed a penicillin rash, three cases having sulphonamides had severe nausea and depression. Three patients were given penicillin lozenges in addition and one developed severe glossitis and stomatitis, so the use of these was discontinued and these three cases not included in this series. But no side effects in this series were severe enough to warrant stopping treatment.

All were kept in bed until well. In order to overcome the possible effect of people not wanting to return to work, at first most attention was paid to clinical signs in pronouncing the patient well. But in fact little evidence of "shirking" was found, and in all cases symptoms and signs resolved together.

The average time taken for recovery is shown in Table III.

TABLE II

| Treatment | Number of cases treated | Average time for recovery |
|---|---|---|
| Symptomatic | 22 | 6½ days |
| Penicillin | 16 | 6⅓ days |
| Sulphonamides | 14 | 6½ days |
| Penicillin and Sulphonamides combined | 8 | 6 days |

The effect of the treatment on the principal symptoms and the temperature was also studied as shown in Table II.

TABLE III

| Treatment | Average time sore throat and dysphagia lasted | Average time temperature lasted |
|---|---|---|
| Symptomatic | 5¼ days | 3 days |
| Penicillin | 5 days | 3 days |
| Sulphonamides | 4¼ days | 3½ days |
| Penicillin and Sulphonamides combined. | 4¼ days | 2½ days |

Complications were few in this small series and are shown in Table IV.

TABLE IV

| Complication | *Number of cases developing the complication on*: Symptomatic Treatment | Penicillin | Sulphonamides | Sulphonamide and Penicillin combined |
|---|---|---|---|---|
| Peritonsillar Abscess | 2 | 0 | 0 | 0 |
| Otitis Media | 1 | 1 | 0 | 0 |
| Generalized Adenitis | 0 | 0 | 1 | 0 |
| Persistent pyrexia with no clinical signs. | 0 | 0 | 1 | 0 |
| Persistent Ulcer of Fauces | 1 | 0 | 0 | 0 |
| Total number of cases treated | 22 | 16 | 14 | 8 |

The cases of peritonsillar abscess resolved on penicillin without surgery. Follow-up of these cases six months later showed no gross evidence of chronic tonsillar disease in any case.

### DISCUSSION

The aim of this investigation was to see if one was justified in treating cases of acute follicular tonsillitis with antibiotics in the absence of evidence of the nature of the causal organism. No throat swabs or blood counts were done for this reason, and the cases were simply identified as acute follicular tonsillitis and all had similar clinical appearances.

The results here show that little is gained by the indiscriminate use of antibiotics and that the times of recovery of the treated and untreated groups are almost the same.

It has recently been established that a condition exists indistinguishable clinically from the tonsillitis caused by the haemolytic streptococci (Commission on Acute Respiratory diseases 1944).[5] This was named "acute endemic exudative pharyngitis and tonsillitis" and is probably a viral infection by one of the adenoido-pharyngeo-conjunctival (A.P.C.) group. These organisms are insensitive to antibiotics and infection by them would be unaffected by antibiotic treatment.

As most cases of acute follicular tonsillitis are treated in General Practice wh[illegible] routine throat swabs are not taken, it is suggested that treatment with antibiotic[illegible] this condition should be withheld unless throat swabs are taken and are organis[illegible] positively identified. Patients infected with haemolytic streptococci will clearly ben[illegible] from penicillin but other causal agents will be unaffected.

As all cases treated in this series present similar appearances clinically it is [illegible] forward as further evidence that the nature of the causal organism cannot be determin[illegible] other than by further investigation (throat swab). It also appears that many of [illegible] cases seen in General Practice will not respond to antibiotics.

### SUMMARY AND CONCLUSIONS

Sixty young men with acute follicular tonsillitis were treated symptomatical[illegible] some were given sulphonamides, penicillin or both drugs in addition. There was [illegible] appreciable difference in the speed of recovery in any of the four groups.

These results indicate that the indiscriminate use of antibiotics in tonsillitis is [illegible] little value.

Streptococcal tonsillitis is impossible clinically to distinguish from those cases [illegible] tonsillitis which do not respond to antibiotics. It is therefore clearly desirable to tak[illegible] swab to identify the causal organism. Where this cannot be arranged routine use of an[illegible] biotics would be indicated in patients who had previously had an attack of ac[illegible] rheumatism.

This work was done whilst holding a temporary commission in the R.A.F. and [illegible] wish to express my thanks to the Air Ministry for permission to publish this pap[illegible]

### *REFERENCES*

[1] Landsman, J. B., Grist, N. R., Black, R., McFarlane, D., Blair, W. and Anderson, T. (19[illegible] *Brit. med. J.*, **1**, 326.

[2] Mcdonald, T. C. and Watson, I. H. (1951). *Brit. med. J.*, **1**, 323.

[3] Denny, F. W., Wannamaker, L. W. and Hahn, E. O. (1953). *Pediatrics*, **11**, 7.

[4] Massell, B. F., Sturgis, G. P., Knobloch, J. D., Streeper, R. B., Hall, T. N. and Norcross, [illegible] (1951). *J. Amer. med. Ass.* **146**., 1469.

[5] Commission on Acute Respiratory Diseases, U.S. Army (1944). *J. Amer. med. Ass*, **125**, 11[illegible]

